# The Effect of Oligofructose-Enriched Inulin on Faecal Bacterial Counts and Microbiota-Associated Characteristics in Celiac Disease Children Following a Gluten-Free Diet: Results of a Randomized, Placebo-Controlled Trial

**DOI:** 10.3390/nu10020201

**Published:** 2018-02-12

**Authors:** Natalia Drabińska, Elżbieta Jarocka-Cyrta, Lidia Hanna Markiewicz, Urszula Krupa-Kozak

**Affiliations:** 1 Department of Chemistry and Biodynamics of Food, Institute of Animal Reproduction and Food Research of Polish Academy of Sciences, Tuwima 10 Str., 10-748 Olsztyn, Poland; n.drabinska@pan.olsztyn.pl; 2 Department of Pediatrics, Gastroenterology and Nutrition, Collegium Medicum, University of Warmia & Mazury, Oczapowskiego 2 Str., 10-719 Olsztyn, Poland; ejarocka@op.pl; 3 Department of Immunology and Food Microbiology, Institute of Animal Reproduction and Food Research of Polish Academy of Sciences, Tuwima 10 Str., 10-748 Olsztyn, Poland; l.markiewicz@pan.olsztyn.pl

**Keywords:** celiac disease, inulin, prebiotic, gluten-free diet, gut microbiota, short-chain fatty acids

## Abstract

Celiac disease (CD) is associated with intestinal microbiota alterations. The administration of prebiotics could be a promising method of restoring gut homeostasis in CD. The aim of this study was to evaluate the effect of prolonged oligofructose-enriched inulin (Synergy 1) administration on the characteristics and metabolism of intestinal microbiota in CD children following a gluten-free diet (GFD). Thirty-four paediatric CD patients (mean age 10 years; 62% females) on a GFD were randomized into two experimental groups receiving Synergy 1 (10 g/day) or placebo (maltodextrin; 7 g/day) for 3 months. The quantitative gut microbiota characteristics and short-chain fatty acids (SCFAs) concentration were analysed. In addition, side effects were monitored. Generally, the administration of Synergy 1 in a GFD did not cause any side effects. After the intervention period, *Bifidobacterium* count increased significantly (*p* < 0.05) in the Synergy 1 group. Moreover, an increase in faecal acetate and butyrate levels was observed in the prebiotic group. Consequently, total SCFA levels were 31% higher than at the baseline. The presented trial shows that Synergy 1 applied as a supplement of a GFD had a moderate effect on the qualitative characteristics of faecal microbiota, whereas it stimulated the bacterial metabolite production in CD children.

## 1. Introduction

Recent data emphasizes the pivotal role of microbiota homeostasis in maintaining the host’s health [1,2]. Commensal bacteria prevent the development of pathologic bacteria in the gut. Additionally, the intestinal microbiome is involved in the differentiation and proliferation of intestinal epithelial cells, the maintenance of gut integrity [1], the synthesis of vital nutrients, in particular vitamins (B-complex vitamins, vitamin K), the absorption of minerals (calcium, magnesium), and the fermentation of food. Therefore, a dysbiotic intestinal microbiota may contribute to the development of gastrointestinal, systemic, and metabolic diseases. It has been reported that disruptions in microbiota composition during infancy may contribute to autoimmune diseases, including celiac disease (CD) [3].

CD is one of the most common chronic and diet-related disorders, affecting approximately 1% of the global population [4]. In predisposed individuals, the ingestion of gluten-containing foods results in autoimmune enteropathy manifested by a variety of intestinal and extra-intestinal symptoms. The role of intestinal microbiota in CD aetiology is unclear, and it remains unknown whether microbiota dysbiosis is the cause or the consequence of CD [5]. Literature data have demonstrated disproportions between Gram-negative and Gram-positive bacteria and a lower number of beneficial strains (*Bifidobacterium*, *Lactobacillus*) in CD patients [6,7]. It has also been suggested that a gluten-free diet (GFD) itself can cause changes in the composition of intestinal bacteria [8,9]. In CD patients, microbiota dysbiosis continues to be observed even after long-term treatment with a GFD [6,10].

Microbiota evaluations can be performed in two ways: by analysing bacterial composition or by analysing bacterial activity [11]. Microbiota-associated characteristics (MAC) are defined as the features of the human body that are influenced by intestinal microbiota [11]. The most widely applied MAC in human studies is the analysis of the profile and concentration of faecal short-chain fatty acids (SCFAs) representing the unabsorbed fraction of SCFAs in the intestinal tract [12]. SCFAs are produced during the fermentation of carbohydrates by intestinal microbiota, and the main products are acetate, propionate, and butyrate. However, if the supply of carbohydrates is limited, the fermentation of other sources of energy, such as proteins and fats, occurs and results in the formation of branched-chain fatty acids (BCFAs) [13].

At present, there are no pharmacological agents against CD; therefore, a GFD is still the only available treatment for CD [14]. The importance of microbiota in CD pathogenesis has shifted the researchers’ focus to the modification of the composition and activity of intestinal microbiota. The administration of probiotics and prebiotics could be proposed as a relatively simple method of improving bacterial composition. The application of certain probiotic bacteria was shown to successfully restore the balance in microbiota composition of CD patients [15]. Prebiotics also confer many beneficial health effects [16], but unlike probiotics they are not living organisms but naturally occurring food components that are selectively utilized by the host microbiota. Among prebiotics, inulin-type fructans are the most widely studied, and are proven to increase *Bifidobacterium* and *Lactobacillus* counts in faecal samples [17]. Prebiotics have been successfully applied to restore the microbiota balance in patients suffering from inflammatory intestinal diseases [18,19], but to date they have not been evaluated in CD patients. Therefore, the aim of this study was to evaluate the effect of oligofructose-enriched inulin on the characteristics and activity of intestinal microbiota in CD children following a GFD.

## 2. Materials and Methods

### 2.1. Study Protocol

Thirty-four children (aged 4 to 18 years; 62% females) diagnosed with CD and following a GFD for at least 6 months were randomly allocated to a placebo (*n* = 16) or a Synergy 1 (*n* = 18) group, according to the study protocol described in detail by Krupa-Kozak et al. [20]. The demographic and anthropometric characteristics of patients have been presented previously [20]. During the 3-month intervention, children of the placebo group consumed maltodextrin (7 g orally/day; Maltodextrin DE 20, Hotrimex, Konin, Poland), whereas children of the Synergy 1 group consumed oligofructose-enriched inulin (10 g orally/day; Orafti^®^ Synergy 1, Beneo, Tienen, Belgium) as a supplement of a GFD. Maltodextrin was selected as a placebo, as it is digested in the small intestine, contrary to prebiotics that reach the colon in intact form. Participants were asked to record the daily compliance with treatment as well as any adverse reactions occurring during the whole trial in the observation chart provided to each participant at the first visit. In addition, they filled out the authors’ original questionnaire concerning the last week of the study, which was aimed at assessing in detail any side-effects after the intake of supplement, such as discomfort, abdominal pain, and stool frequency and consistency. The last week was selected because the effect of the prebiotic was the most dominant at this stage of the study.

### 2.2. Ethics

The participants’ parents and caregivers signed written consent forms during the enrolment visit. The experimental design and all procedures have been approved by the Bioethics Committee of the Faculty of Medical Sciences of the University of Warmia and Mazury in Olsztyn (permission No. 23/2015 of 16 June 2015). Study visits were coordinated with regular visits held every 3 months in a gastroenterology clinic. The study was registered at ClinicalTrials database under the number NCT03064997 [21].

### 2.3. Samples Collection

The details of sample collection were presented in Krupa-Kozak et al. [20]. Briefly, faecal samples were collected from patients (at the baseline and at the end of experiment), transported to a laboratory on ice, and aliquoted separately for bacterial DNA isolation and SCFA analysis. For the genomic assay, approximately 100 mg of faeces was separated and stored at −80 °C. For the SCFA analysis, two aliquots of approximately 100 mg were weighted, and 150 μL of 10% formic acid was added to each sample as preservative before storage at −80 °C.

### 2.4. Isolation of Bacterial Genomic DNA

Immediately before DNA isolation, samples were defrosted at room temperature. For a quantitative analysis of microbial characteristics, genomic bacterial DNA was isolated from faecal samples using a commercial kit designated for human faecal specimens (GeneMATRIX Stool DNA Purification Kit, EURx, Gdańsk, Poland) according to the manufacturer’s protocol using the bead-beating method and a Gyrator UNIPREP 3D vortex (UniEquip, Planegg, Germany) as described in [22].

### 2.5. Quantitative Real-Time PCR

The quantitative characteristics of intestinal bacteria were determined by the real-time polymerase chain reaction (RT-PCR) technique with the use of group-specific primers according to the procedure described by Fotschki et al. [22] The DNA of predominant bacterial strains was used as a positive control and for the preparation of standard curves. Pure cultures of bacterial strains were cultivated as described by Fotschki et al. [22] and DNA was isolated with the same method as applied for faecal samples. Total bacterial counts and the counts of *Bifidobacterium* (BIF) and *Lactobacillus* (LAC) genera, the *Bacteroides-Prevotella-Porphyromonas* (BPP) group, and the *Clostridium coccoides* (Ccocc) and *Clostridium leptum* (Clept) groups were determined. Decimal dilutions of the DNA standard were used to plot a standard curve with each primer pair (Table 1). To be able to compare samples to the standards, the extracted DNA was not normalized to keep the same isolation efficiency and amplification conditions, allowing for the quantitative analysis. Amplifications were performed in the Quant Studio 6 Flex real-time PCR system (Thermo Fisher, Warsaw, Poland) in a total volume of 20 μL (10 μL of SYBR Green Jump-Start Taq ReadyMix (Sigma-Aldrich, Saint Louis, MO, USA), 1 μL of 10-fold diluted DNA, 200 μM of each primer, and PCR-grade water). The temperature program was as follows: 1 cycle at 95 °C for 2 min, 40 cycles at 95 °C for 15 s, primer annealing temperature (Table 1) for 1 min, and 72 °C for 30 s with signal acquisition. After each run, a melting curve was prepared to confirm the specificity of amplicons. The values were normalized according to the dilution and weight of the sample. The analyses were performed in duplicate, and final data were expressed as log10 of bacterial cells per gram of the sample on a wet weight basis.

### 2.6. Microbiota-Associated Characteristics

Individual SCFAs in fecal samples were analysed by gas chromatography according to a modified method of Garcia-Villaba et al. [28]. Briefly, faecal samples of approximately 100 mg and 500 μL of a mixture of acetone and 0.03 M oxalic acid (3:2 ratio) were homogenized with a vortex for 1 min. Acetone was found to better extract SCFA than all of the solvents proposed by the authors [28]. Dilution with water gave unsatisfactory peak shapes; therefore, it was replaced by aqueous 0.03 M oxalic acid, which hardly improved peak shapes and allowed us to obtain higher levels of SCFA [29]. Then, samples were centrifuged for 20 min at 13,400 rpm, and the supernatants were collected into chromatography vials and introduced to the gas chromatography (GC) system.

The SCFAs were analysed in the Agilent 7890A gas chromatograph (Agilent, Wilmington, DE, USA) with a flame-ionization detector (FID) and the 7683B auto-injector. The compounds were separated in the SGE BP21 capillary column (SGE Analytical Science by Trajan, Ringwood, VIC, Australia; 30 m × 0.53 mm, film thickness: 0.5). The carrier gas was helium (Air Products and Chemicals, Allentown, PA, USA; 5 mL/min). The samples (0.5 µL) were injected in a split mode (10:1). The oven temperature was initially set at 85 °C, raised to 180 °C (6 °C/min), and maintained for 5 min. The injector temperature was 250 °C and the temperature of the flame ionization detector was 290 °C. External standards of acetic, propionic, butyric, isobutyric, valeric, and isovaleric acid were applied. The analyses were performed in triplicate.

### 2.7. Statistical Analysis

The results of the quantitative analyses of faecal microbiota and SCFAs are expressed as means ± standard error of the mean (SEM). The data collected from subjects with reported 80% intake were used in the final analysis (*n* = 25). Normal distribution of data was evaluated by the Shapiro–Wilk test. Student’s *t*-test or the Mann–Whitney *U* test was applied to compare differences between groups, as appropriate. Differences within groups before and after intervention were determined with the Student’s *t*-test or the Wilcoxon test, as appropriate. All statistical analyses were conducted using Statistica v. 12 software (StatSoft, Krakow, Polska). The significance of differences between the samples was determined at *p* < 0.05.

## 3. Results

### 3.1. Response to Intervention

In general, Synergy 1 was well-accepted by trial participants and did not cause discomfort. Based on three-months of observation, no specific side-effects were observed, except for single episodes of abdominal pain or diarrhoea that were noticed occasionally in both experimental groups. In one child suffering from bloating and constipation before the enrolment, the alleviation of symptoms during the intake of Synergy 1 was noticed. The results of detailed observations of the last intervention week are summarised in Table 2. Mild abdominal pain lasting for two subsequent days was reported in three children, both in the Synergy 1 and placebo groups. The defecation frequency was similar in both experimental groups; however, the consistency of stool in children on a GFD supplemented with Synergy 1 was usually described as normal (95% of the time), while in the placebo group the consistency of stool was perceived as normal only 69% of the time. Two participants reported discomfort after taking the placebo that was described as moderate nausea.

### 3.2. Quantitative Profile of Fecal Microbiota

**Figure 1:**
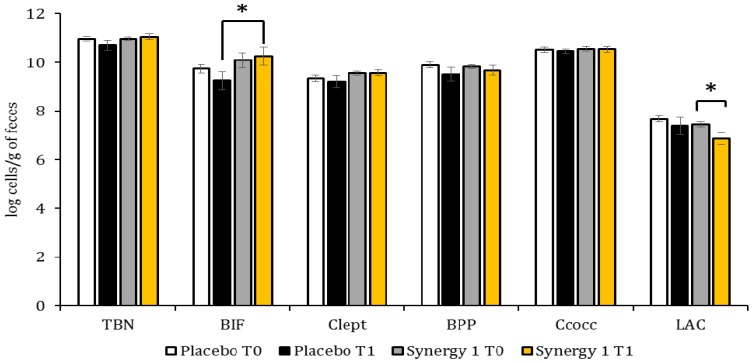
Quantitative characteristics of faecal microbiota before (T0) and after (T1) the intervention, expressed by means ± SEM (standard error of the mean) and by log cells/g of faeces. * *p* < 0.05. TBN: total bacterial counts; BIF: *Bifidobacterium* sp.; Clept: *C. leptum*; BPP: *Bacteroides-Prevotella-Porphyromonas*; Ccocc: *Clostridium coccoides*; LAC: *Lactobacillus* sp.

The quantitative profiles of faecal microbiota of CD children are presented at Figure 1. The initial total bacteria number (TBN) as well as the initial counts of individual bacteria were similar in both experimental groups. A comparison of bacteria counts between the experimental groups after the three-month intervention revealed a significant increase (*p* < 0.05) in BIF count in the Synergy 1 group. Moreover, the count of Clept in the Synergy 1 group was stable and at the end of experiment it was similar to the initial count, whereas in the placebo group the final Clept count was reduced. After the intervention, a decrease in the count of LAC was noticed in both experimental groups, but it was significant only in the Synergy 1 group. The determined alterations in the quantity of individual bacteria resulting from the intervention were reflected by the TBN, which remained constant in the Synergy 1 group, but tended to decrease in the placebo group.

### 3.3. Microbiota-Associated Charcteristics

Based on the results of the gas chromatography analysis, acetic, propionic, and butyric acid were determined in dominant concentration in faecal samples of CD children (Table 3). Initially, the concentration of individual SCFAs was statistically similar in both experimental groups. After the experiment, a significant (*p* < 0.05) increase in faecal acetate and butyrate levels was observed in the group receiving Synergy 1; consequently, total SCFAs concentration was 31% higher than at the baseline. A comparison of both groups after the intervention revealed significantly (*p* < 0.05) higher levels of acetate and propionate in the Synergy 1 group. At the end of experiment, the faecal concentration of total SCFAs in the Synergy 1 group was significantly (*p* < 0.05) higher than in the placebo group.

The initial concentrations of total BCFAs, expressed as a sum of *iso*-butyric and *iso*-valeric acids, were similar in both experimental groups (Table 3). After the intervention, the relative concentration of BCFAs, expressed as the percentage of the total BCFAs in the total SCFAs, decreased by 31% in the Synergy 1 group but increased by 15% in the placebo group.

## 4. Discussion

Inulin-type fructans are one of the most widely researched groups of prebiotic compounds [30]. Their positive effect in counteracting intestinal microbiota dysbiosis has been demonstrated in human studies [31]. To the best of our knowledge, prebiotics have never been applied as a GFD supplement in CD patients. This study makes the first attempt to evaluate the effect of inulin-type fructans on gut microbiota characteristics and activity in CD children.

The first finding to emerge from the present study was an increase in the count of BIF in children following a GFD supplemented with Synergy 1. Our results are consistent with published studies where inulin-type fructans had a stimulating effect on BIF species regardless of the applied dose, degree of polymerization, or the duration of the experiment [32,33]. In CD patients, both adults and children, a decrease in the counts and diversity of BIF species have been reported [8]. Taking this into account, the obtained results are promising as they indicated that the administration of Synergy 1 in a GFD may counteract the reduction in BIF count in CD children. Much research has pointed out health-associated benefits of BIF and demonstrated the stimulation of immune response and suppression of pro-inflammatory cytokine synthesis [34,35,36]. However, it is important to highlight that changes in microbiota resulting from a dietary intervention with a prebiotic depend among other factors on the initial microbiota composition [37]. Tuohy et al. [38] observed a significant increase in BIF count in healthy volunteers receiving 8 g of inulin for two weeks, but a more prominent increase was observed in subjects with lower initial bacterial counts. After analysing the count of other bacteria groups, a constant quantity in Clept was observed in the experimental group supplemented with Synergy 1, whereas a decreasing trend was observed in the placebo group. Clept is one of the predominant groups of gut bacteria, with a 16–25% share of the total bacteria [39], and includes *Faecalibacterium prausnitzii* and selected species of *Eubacterium* and *Ruminococcus* [40]. Clept plays a vital role in the intestine. It is involved in the fermentation of unabsorbed carbohydrates, which leads to the formation of butyric acid [41]. Changes in the composition and reduced counts of Clept group bacteria are characteristic of inflammatory intestinal diseases [42]. Dietary intervention with the prebiotic Synergy 1 resulted in a decrease in the final LAC count, which was an unforeseen result as based on literature data, inulin-type fructans were expected to stimulate the growth of LAC species [32]. In contrast, Adebola et al. [43] demonstrated that inulin did not stimulate any of the five probiotic strains of LAC, while other potential prebiotics, including lactulose and lactobionic acid, were utilized by bacteria and minimized the adverse effects of bile acid stress. The cited authors concluded that LAC have potentially stimulating effects only in specific formulations of probiotics and prebiotics [43]. A previous animal study on rats fed a GFD [44] showed that diets containing inulin reduced the faecal LAC count.

Although oligofructose-enriched inulin applied as a supplement of a GFD had a rather moderate effect on changes in the qualitative characteristics of faecal microbiota of CD children, it impacted considerably the bacteria activity and altered the amount of bacterial metabolites. We noticed a remarkable increase in the total SCFAs concentration in the Synergy 1 group, mainly due to a significant increase in acetate concentration. This result could be attributed to an effective fermentation of inulin-type fructans administered in a GFD, which are a readily available source of energy for gut microbiota. Our findings are in agreement with previous studies reporting that diet and dietary compounds can modulate the metabolic activity of the intestinal microbiota rather than its quantitative and qualitative composition [45,46]. On the other hand, it could be expected that the acidification of the intestinal environment as a result of excessive SCFAs production could be associated with changes in bacteria composition, and potentially may promote the growth of beneficial microbiota simultaneously reducing the number of pathogenic species. In the present study, the increase in SCFAs amount was accompanied with the rise of BIF quantity. Protective BIF are considered to be the main producers of acetate [47]. Previously, acetic acid has been regarded as a pro-inflammatory trigger [48] but recent studies have demonstrated that acetate can prevent enteropathogenic infections [47]. The concentration of acetic acid is correlated with the expression of the Apoe, C3, and Pla2g2a genes, which are involved in the anti-inflammatory response [47]. Moreover, acetate prevents *Escherichia coli* O157-induced apoptosis of epithelial cells by decreasing transepithelial electrical resistance, and it is also involved in the reduction of intestinal permeability [47]. Acetate has been detected in the circulation at a concentration of 50–150 μM, which could point to a systemic mechanism of microbiota regulation [49]. The increase in acetic acid had the most dominant effect on the increase of total SCFAs. Consequently, the relative content of BCFAs was reduced after intervention, even though the concentration of BCFAs remained unchanged.

The concentrations of propionate and butyrate also increased in subjects supplemented with Synergy 1. The above could be attributed to the formula of Synergy 1, which is a mixture of short-chain fructooligosaccharides and long-chain inulin. Butyric acid is the main fermentation product of oligosaccharides, whereas inulin fermentation leads to the formation of propionic acid [50]. The importance of the increase in butyrate levels is associated with its role in the proliferation and differentiation of colon epithelial cells. Butyric acid regulates tight junction proteins and improves the integrity of the gut barrier by stimulating the expression of claudin-1 and zonula occludens-2, which participate in tight-junction assembly [13]. Butyric acid has also been shown to alleviate gastrointestinal mucosal inflammation [51].

## 5. Conclusions

In conclusion, the results of this study have demonstrated that the administration of oligofructose-enriched inulin is harmless and modulated the proportion of bacterial species in faecal samples, increasing the counts of BIF in paediatric CD patients adhering to a GFD. However, the main findings were related to the shifting the faecal concentrations of bacterial metabolites provoked by prebiotic administration. In this study, the addition of Synergy 1 to a GFD of CD children modified the faecal SCFA profile, in particular increasing the concentration of acetate. This is the first-ever study to evaluate the effect of prebiotics on the gut microbiota of children with CD. Our findings indicate that GFD supplementation with Synergy 1 could be a promising therapeutic approach for modulating intestinal microbiota and their activity. Moreover, taking into account the critical role of intestinal microbiota in the development of several autoimmune diseases, such as type 1 diabetes [52], the application of prebiotics in a GFD may be helpful in the prevention of and protection against such possible complications. Admitting that the study reported herein has some limitations, in particular a small population size, inclusion of children with a wide age-range, and a relatively short intervention duration, the findings are interesting enough to encourage further well-powered intervention studies, which would provide sound data on the efficacy of prebiotic administration in the diet of CD patients.

## Figures and Tables

**Table 1:** The primers used in the real-time polymerase chain reaction (RT-PCR) analysis.

Target	Primer	Sequence (5′ → 3′)	T_a_ (°C)	Reference
Total bacteria	UniF	GTGSTGCAYGGYYGTCGTCA	60	[23]
	UniR	ACGTCRTCCMCNCCTTCCTC		
*Bifidobacterium*	BIF-F	TCG CGTC(C/T)GGTGTGAAAG	58	[24]
	BIF-R	CCACATCCAGC(A/G)TCCAC		
*Clostridium leptum* group	sg-Clept-F	GCACAAGCAGTGGAGT	53	[25]
	sg-Clept-R3	CTTCCTCCGTTTTGTCAA		
*Bacteroides-Prevotella-Porphyromonas*	BPP-F	GGTGTCGGCTTAAGTGCCAT	56	[24]
	BPP-R	CGGA(C/T)GTAAGGGCCGTGC		
*Clostridium coccoides* group	g-ccoc-F	AAATGACGGTACCTGACTAA	58	[26]
	g-ccoc-R	CTTTGAGTTTCATTCTTGCGAA		
*Lactobacillus*	Lac1F	AGCAGTAGGGAATCTTCCA	58	[27]
	Lab667	CACCGCTACACATGGAG		

**Table 2:** Last-week survey observation.

Observation	Synergy 1	Placebo
Abdominal pain ^1^	3	3
Everyday defecation ^2^ (%)		
Yes	90	81
No	10	19
Defecation No/day	1.3 ± 0.5	1.1 ± 0.2
Normal stool consistency ^3^ (%)	95	69
Discomfort after the intake of supplement	0	2

^1^ Number of episodes; ^2^ during 7-day evaluation period; ^3^ no diarrhea or constipation.

**Table 3:** Short chain fatty acids (SCFAs) concentration in faecal samples before (T0) and after (T1) the intervention, expressed by means ± SEM and by μmol/g of faeces.

SCFA	Placebo Group		Synergy 1 Group		Placebo: T0 vs. T1 ^2^	Synergy 1: T0 vs. T1	T1: Placebo vs. Synergy 1 ^3^
	T0	T1	T0	T1			
Acetic	29.84 ± 1.93	28.82 ± 4.26	30.00 ± 3.35	44.06 ^1^ ± 4.13	0.520	0.047 *	0.020 *
Propionic	9.91 ± 1.23	7.97 ± 1.55	10.16 ± 1.25	11.14 ± 0.61	0.692	0.328	0.048 *
*Iso*-butyric	1.41 ± 0.15	1.56 ± 0.33	1.60 ± 0.14	1.33 ± 0.13	0.678	0.059	1.000
Butyric	8.92 ± 1.37	8.21 ± 1.89	8.02 ± 1.27	10.41 ± 0.89	0.889	0.047 *	0.292
*Iso*-valeric	1.43 ± 0.18	1.46 ± 0.23	1.72 ± 0.19	1.56 ± 0.16	0.515	0.400	0.419
Valeric	1.55 ± 0.21	0.97 ± 0.16	1.72 ± 0.18	1.47 ± 0.18	0.030 *	0.123	0.062
Total SCFAs	53.06 ± 5.52	48.99 ± 5.22	53.23 ± 6.94	69.95 ± 5.92	0.418	0.041 *	0.005 *
Total BCFAs	2.84 ± 0.30	3.02 ± 0.40	3.32 ± 0.33	2.89 ± 0.28	0.765	0.132	0.679
Relative BCFAs ^1^	5.35	6.16	6.24	4.13			

* *p* < 0.05. ^1^ Relative concentration of the total branched-chain fatty acids (BCFAs) (sum of *iso*-butyic and *iso*-valeric acids) expressed as a percentage of the total SCFA. ^2^ Comparison between groups using Student’s *t*-test or the Mann–Whitney *U* test, as appropriate. ^3^ Comparison within groups before and after intervention using the Student’s *t*-test or the Wilcoxon test, as appropriate.
